# *LcCOL7* and *LcCOL8* Negatively Regulate Plant Flowering Independent of Day Length

**DOI:** 10.3390/plants15142139

**Published:** 2026-07-10

**Authors:** Tingting Yan, Yukun He, Tianyi Tang, Haida Deng, Ding Chen, Farhat Abbas, Zhe Chen, Mingchao Yang, Xianghe Wang, Fuchu Hu

**Affiliations:** 1 Institute of Tropical Fruit Trees, Hainan Academy of Agricultural Sciences/Key Laboratory of Genetic Resources Evaluation and Utilization of Tropical Fruits and Vegetables (Co-Construction by Ministry and Province), Ministry of Agriculture and Rural Affairs/Key Laboratory of Tropical Fruit Tree Biology of Hainan Province, Haikou 571100, China; yantt_hnaas@163.com (T.Y.); heyukun196@163.com (Y.H.); tangtianyi1518@163.com (T.T.); denghaida2000@163.com (H.D.); cd93_hnaas@163.com (D.C.); farhatabbas@scau.edu.cn (F.A.); chenzhe@hnaas.org.cn (Z.C.); yangmc_hnaas@163.com (M.Y.); wangxh198@126.com (X.W.); 2 Sanya Institute of China Agricultural University, Sanya 572025, China; 3 Sanya Research Institute, Hainan Academy of Agricultural Sciences, Sanya 572025, China

**Keywords:** litchi, CONSTANS, photoperiod, circadian, flowering

## Abstract

*CONSTANS-LIKE* (*COL*) genes are pivotal regulatory components in the photoperiodic flowering pathway of plants. These genes can be regulated by both photoreceptors and the circadian clock, modulating plant flowering responses under specific day lengths by regulating florigen levels. However, the *COL* gene family in *Litchi chinensis* Sonn. has not yet been characterized. In this study, we identified eight *COL* family members in litchi and classified them into three subgroups based on phylogenetic analysis. The analysis of *cis*-regulatory elements within the promoters of *LcCOLs* revealed a wide distribution of elements associated with light, hormone, and stress responses. Transcript expression profiling indicated that most *LcCOLs* exhibited relatively high expression levels in leaf buds, leaves, and young fruits. Diurnal expression analysis under natural photoperiod conditions revealed that the expression peaks of all *LcCOLs*, with the exception of *LcCOL1*, occurred during the nighttime. The heterologous overexpression of *LcCOL7* and *LcCOL8*, the closest homologs to *AtCO*, in *Arabidopsis thaliana* significantly delayed the flowering time under both long-day (LD) and short-day (SD) conditions, indicating that these genes act as repressors of flowering. This study provides a foundational basis for elucidating the molecular mechanisms underlying litchi flowering regulation and identifies promising candidate genes for the molecular breeding of litchi flowering-related agronomic traits.

## 1. Introduction

Floral transition is a critical developmental process for plant reproduction, which is co-regulated by multiple pathways including temperature, day length (photoperiod), gibberellins and plant age [1,2,3]. Within the photoperiodic flowering pathways, the CONSTANS (CO) protein serves as a central regulator and is ubiquitously present across diverse plant species. For instance, *Arabidopsis thaliana* contains 17 members of the *CONSTANS-LIKE* (*COL*) family, including *CO*, while 16 *COL* genes have been identified in *Oryza sativa* L., nine in *Hordeum vulgare*, 33 in *Brassica napus*, nine in *Fragaria vesca* L., 15 in *Pyrus bretschneideri* and 10 in *Dimocarpus longan* Lour [4,5,6,7,8]. *COL* family members in different plant species participate in the regulation of floral transition, with some promoting flowering [6,9,10,11], and others functioning as repressors [7,8,12,13]. This highlights the crucial role of *COL* family genes in plant floral development and their significant contribution to the precise regulation of flowering in response to varying environmental conditions.

Based on their flowering behaviors in response to varied photoperiods, plants can be categorized into three groups: long-day (LD), short-day (SD) and day-neutral (DN) plants. LD plants flower when the day length exceeds the critical day length, whereas SD plants flower when the photoperiod is shorter than the critical threshold [14,15]. Plants insensitive to photoperiod are defined as DN plants. As master regulators, COL proteins integrate photoperiod signals downstream of photoreceptors and the circadian clock [14,15,16], modulating the expression of florigen gene *FLOWERING LOCUS T* (*FT*) to govern photoperiod-dependent flowering. After being synthesized in leaves under flower-inductive conditions, florigen moves to the shoot apical meristem to initiate floral transition [17]. In *Arabidopsis*, the model LD plant, the diurnal transcription pattern of *CO* is governed by the phase and activity of FLAVIN-BINDING KELCH REPEAT F-BOX 1 (FKF1) and GIGANTEA (GI) [14,18]. At dusk under LD conditions, FKF1 is activated by blue light and interacts with GI [18]. This complex promotes the degradation of CYCLING DOF FACTORs (CDFs), which function as repressors of *CO*, and consequently facilitates *CO* transcription. In darkness, the CO protein is degraded by the ubiquitin ligase complex composed of CONSTITUTIVE PHOTOMORPHOGENIC1 (COP1) and SUPPRESSOR OF PHYTOCHROME A (SPA1), while CO protein remains stable in the presence of light [19,20]. Such multi-layered regulatory mechanisms enable CO to precisely perceive ambient photoperiod signals, ensuring timely flowering in *Arabidopsis*. *Oryza sativa* serves as a model SD plant, with its photoperiodic flowering also regulated by the CO-FT homologous pathway [14,15]. In rice, *Heading date 3a* (*Hd3a*), an ortholog of the *FT* gene, accelerates floral transition under inductive SD conditions, while *RICE FLOWERING LOCUS T 1* (*RFT1*) is required for flowering under non-inductive LD conditions [21,22]. *Heading date 1* (*Hd1*), the ortholog of *CO*, predominantly accumulates at night under SD conditions and activates *Hd3a* expression to trigger flowering [22,23]. Under non-inductive LD conditions, *Hd1* expression persists from nighttime to dawn; upon light-dependent accumulation, Hd1 acts as a transcriptional repressor of *Hd3a* via PHYTOCHROME B (PHYB)-mediated red light signaling, consequently functioning as a flowering suppressor [14,24,25]. Collectively, these examples demonstrate that COL homologs mediate photoperiod perception to control flowering across diverse plant species, yet their underlying regulatory mechanisms differ markedly between taxa.

COL proteins feature two characteristic conserved domains: an N-terminal B-box domain and a C-terminal CONSTANS, CO-LIKE and TOC1 (CCT) motif [16,26,27,28]. The B-box domain is a zinc-finger module mainly responsible for mediating protein–protein interactions [16,27,28]. According to the copy number and sequence conservation of B-box domains, COL family proteins are divided into three subclades. Group I contains two canonical B-box domains; Group II harbors a single B-box; Group III carries one conserved B-box plus one divergent B-box variant [26]. The CCT domain contains a nuclear localization signal and also participates in protein–protein interactions [16,29]. For example, AtCO interacts with NUCLEAR FACTOR Y B SUBUNIT/NUCLEAR FACTOR Y C SUBUNIT (NF-YB2/NF-YC3) to form a trimeric complex, which promotes complex binding to the CORE regulatory motif in the promoter of *FT* [16,29]. Mutations disrupting conserved residues in either the B-box or CCT domain of AtCO result in late-flowering phenotypes, demonstrating that both domains are essential for CO-dependent flowering regulation [28].

Pioneering research exploring photoperiodic regulation of litchi (*Litchi chinensis* Sonn.) flowering can be traced to 1966. Using the litchi cultivar ‘Kwai Mi’ as plant material, Nakata et al. [30] observed no obvious difference in flowering onset between LD (16 h light/8 h darkness) and SD (8 h light/16 h darkness) treatments, leading them to classify litchi as a DN species. Even so, accumulating recent evidence implies that the photoperiod pathway participates in modulating litchi floral transition. Genomic screening of 72 litchi accessions with contrasting ripening seasons identified two *LcCOL* genes as candidate loci linked to fruit maturation date, a trait largely governed by flowering initiation time [31]. Furthermore, multiple litchi photoperiod/circadian clock genes such as *FKF1*, *CO*, *COP1*, *PHYC* and *PSEUDORESPONSE REGULATOR 9* (*PRR9*) have been identified as chilling accumulation-associated candidate genes, whose expression differs between early- and late-flowering accessions [32]. These observations suggest that the photoperiod pathway works synergistically with low-temperature signaling to modulate litchi flowering induction.

As central regulators within the photoperiodic flowering pathway, the *COL* gene family has not been systematically characterized in litchi, and their roles in floral development remain poorly understood. In this study, we identified eight *LcCOL* family members in litchi and classified them into three distinct clades based on phylogenetic analysis. We subsequently characterized their conserved motifs and gene structures, and profiled their expression across various litchi tissues as well as under natural photoperiod conditions. Functional assays via heterologous overexpression in *Arabidopsis* further demonstrated that both LcCOL7 and LcCOL8 markedly repress flowering under both LD and SD conditions. Collectively, this study fills the research gap regarding the *COL* gene family in litchi, and provides theoretical insights into the molecular mechanisms controlling litchi flowering.

## 2. Results

### 2.1. Identification of LcCOL Family Genes in Litchi

**Figure 1:**
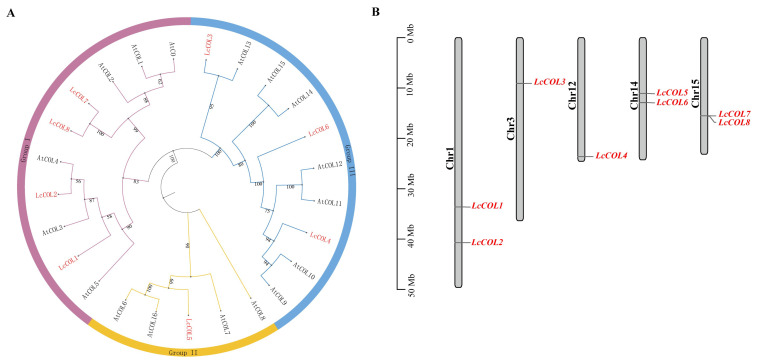
Phylogenetic analysis and chromosomal distribution of the *CONSTANS-LIKE* (*COL*) gene family in litchi. (**A**) Phylogenetic tree of COL proteins from *Litchi chinensis* Sonn. and *Arabidopsis thaliana*. Bootstrap values were inferred from 1000 replicates. (**B**) Chromosomal localization of eight *LcCOL* genes in the litchi genome.

To systematically identify the members of the litchi *LcCOL* gene family, we adopted a two-step screening strategy combining BLASTp searches and InterPro database annotation. This screening pipeline yielded eight *LcCOL* genes, which were designated *LcCOL1* to *LcCOL8* based on their chromosomal distribution (Figure 1). Table 1 summarizes the key physicochemical properties of these eight LcCOL proteins. Their amino acid lengths vary from 347 to 499 residues, with molecular weights ranging from 37.6 kDa to 56.2 kDa. The isoelectric points (pIs) fall between 5.11 and 5.94, and their instability indices (IIs) range from 34.98 to 54.79. Their aliphatic indices (AIs) span from 56.02 to 65.99; most values are lower than 65, indicating generally low thermal stability for LcCOL proteins. Grand average of hydropathy (GRAVY) values were calculated to be between −0.838 and −0.307, demonstrating that all LcCOL proteins are hydrophilic.

Phylogenetic reconstruction utilizing AtCO and AtCOL protein sequences clustered the eight LcCOL members into three distinct subgroups (Figure 1A). Group I comprises LcCOL1, LcCOL2, LcCOL7, and LcCOL8, which form a clade that is closely related to AtCO and AtCOL1-AtCOL5. LcCOL5 falls into Group II together with AtCOL6-AtCOL8 and AtCOL16. The remaining three members, LcCOL3, LcCOL4, and LcCOL6 were assigned to Group III, alongside AtCOL9-AtCOL15.

### 2.2. Conserved Motifs and Gene Structure Analysis of LcCOL Members

**Figure 2:**
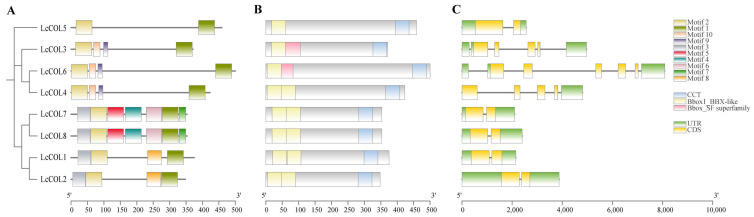
Schematic illustration of conserved motifs, conserved protein domains and gene structures of *LcCOL* family members. (**A**) Distribution of conserved motifs across LcCOL proteins; (**B**) conserved domain architecture of LcCOL proteins; (**C**) exon–intron gene structure of individual *LcCOL* genes.

Conserved motif analysis of LcCOL proteins (Figure 2A) demonstrated that the Group I paralogs LcCOL7 and LcCOL8, the closest phylogenetic homologs of AtCO, possess the largest set of conserved motifs, namely motifs 1–5, 7, and 9. The other two Group I members, LcCOL1 and LcCOL2, contain motifs 1, 2, 3, and 8. All three Group III members (LcCOL3, LcCOL4, and LcCOL6) contain motifs 1, 2, 9, and 10. In contrast, the single Group II protein LcCOL5 only retains motifs 1 and 2. Protein domain analysis (Figure 2B) indicated that proteins from Groups I and III each carry two B-box domains and one CCT domain, while the Group II protein only contains one B-box domain plus one CCT domain. These structural features strongly validate our phylogenetic grouping results. Furthermore, gene structure characterization (Figure 2C) revealed that *LcCOL1*, *LcCOL2*, *LcCOL5*, *LcCOL7*, and *LcCOL8* possess simple gene architectures composed of two exons separated by a single short intron. In contrast, *LcCOL3*, *LcCOL4*, and *LcCOL6* harbor multiple relatively long introns.

### 2.3. Analysis of cis-Regulatory Elements in the Promoter Sequences of LcCOL Genes

**Figure 3:**
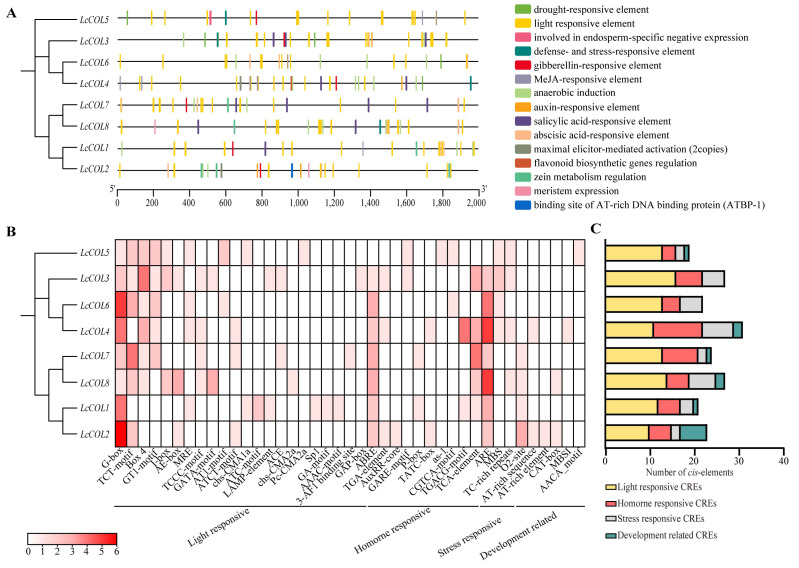
Analysis of *cis*-regulatory elements in the promoter regions of *LcCOL* genes. (**A**) Genomic distribution of diverse *cis*-regulatory elements identified in *LcCOL* promoters; (**B**) heatmap illustrating the abundance of distinct *cis*-elements across individual *LcCOL* gene promoters; (**C**) statistical quantification and classification of identified *cis*-elements in *LcCOL* gene promoters.

We systematically analyzed and statistically characterized cis-regulatory elements within the promoter sequences of the eight *LcCOL* family genes to decipher their transcriptional regulation patterns. As summarized in Figure 3 (detailed data listed in Supplementary Tables S2–S5), the promoters of all *LcCOL* members contain an abundance of light-responsive elements, among which the G-box appears most prevalent, followed by the TCT motif and Box 4. Multiple hormone-responsive *cis*-elements were also detected in these promoters, including those that respond to abscisic acid (ABA), gibberellin (GA), jasmonic acid (JA), auxin, and salicylic acid (SA). Of these hormone-related motifs, the ABA-responsive element (ABRE) and SA-responsive element (TCA-element) were present at the highest frequencies. With regard to stress-responsive *cis*-elements, the anaerobic responsive element (ARE) occurred most frequently. Collectively, these findings suggest that the transcription of *LcCOL* genes is coordinately modulated by crosstalk between environmental cues and endogenous phytohormone signaling pathways, which may participate in regulating litchi growth and development.

### 2.4. Analysis of the Expression Patterns of LcCOLs in Different Tissues of Litchi

**Figure 4:**
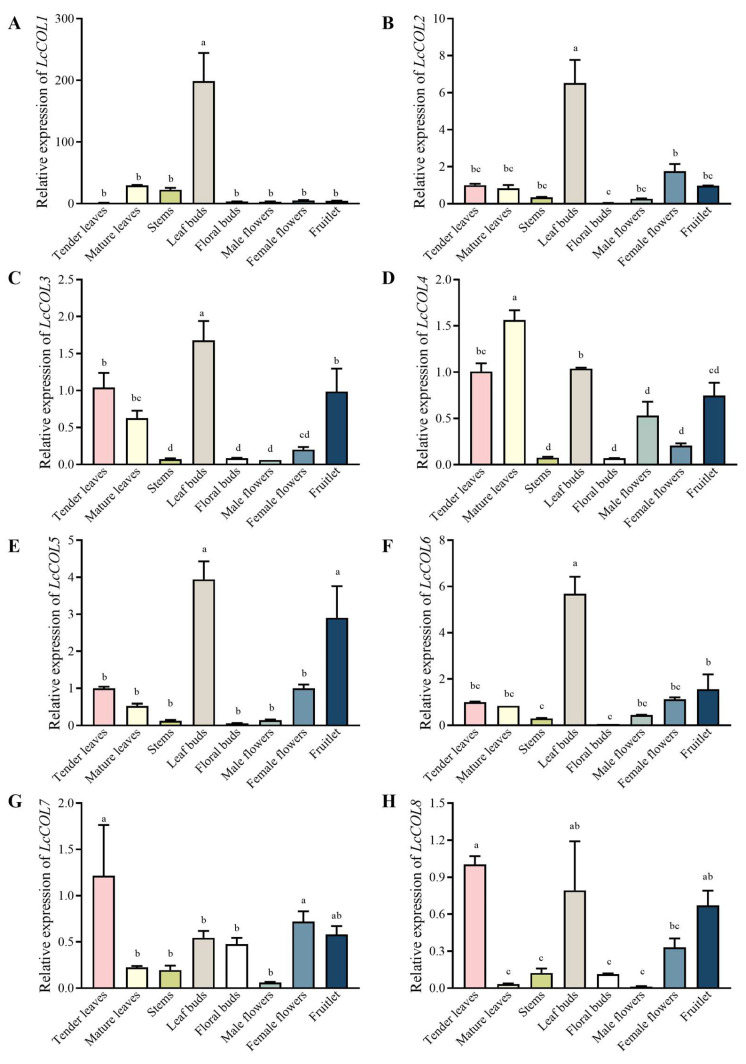
Tissue-specific expression patterns of *LcCOL* genes in litchi. (**A**–**H**) Relative expression levels of individual *LcCOL* genes in different litchi tissues. Tender leaves, mature leaves, leaf buds and stem segments were collected during the vegetative shoot growth stage; flower buds and fully opened male and female flowers were sampled at the flowering stage and young fruitlets were harvested one week after female flowers anthesis. All experiments were performed with three independent biological replicates. Error bars represent standard error of the means (*n* = 3). Different letters above the columns indicate significant differences based on one-way analysis of variance (ANOVA, *p* < 0.05).

To characterize the tissue-specific expression profiles of *LcCOL* genes, we quantified their transcript abundance in eight tissues of the litchi cultivar ‘Feizixiao’, including tender leaves, mature leaves, stems, leaf buds, flower buds, male flowers, female flowers, and fruitlets (Figure 4). The tissue expression patterns revealed that five *LcCOL* members consistently displayed significantly higher expression levels in leaf buds than in other tissues (*p* < 0.05), with the exception of *LcCOL4*, *LcCOL7*, and *LcCOL8,* which exhibited the highest transcript levels in leaf tissues. Dramatic expression differences were also detected between tender and mature leaves. Specifically, *LcCOL4* was significantly more highly expressed in mature leaves (*p* < 0.05), while *LcCOL7* and *LcCOL8* showed preferential expression in tender leaves. The remaining *LcCOL* genes exhibited no obvious expression differences between the two leaf types. All *LcCOL* genes showed relatively low expression levels in stems, another vegetative tissue. In reproductive tissues, moderate transcript levels of *LcCOL7* and *LcCOL8* were detected in floral buds, while other *LcCOL* members were barely detectable. In male flowers, only *LcCOL4* showed weak expression. By contrast, moderate expression of *LcCOL2*, *LcCOL5*, *LcCOL6*, *LcCOL7*, and *LcCOL8* were observed in female flowers. In young fruitlets, all *LcCOL* genes except *LcCOL1* showed a certain degree of expression. Overall, *LcCOL* genes were predominantly expressed in leaf buds, leaves, and fruitlets, with generally low expression in floral tissues. Nevertheless, individual *LcCOL* members exhibited distinct tissue-specific expression patterns, implying divergent biological functions.

### 2.5. Expression Patterns of LcCOLs Under Natural Photoperiod During the Flowering Induction Period

**Figure 5:**
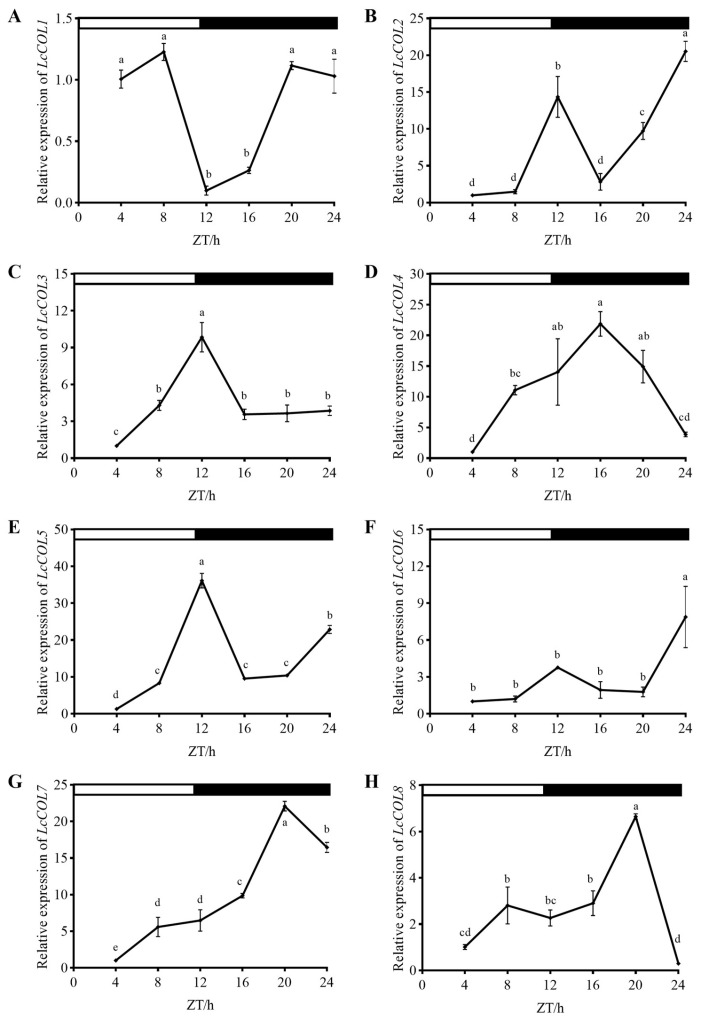
Diurnal expression patterns of *LcCOL* genes under a natural photoperiod. (**A**–**H**) Relative transcript levels of individual *LcCOL* genes under a natural photoperiod with approximately 11 h of light and 13 h of darkness. Error bars represent standard error of the mean (*n* = 3). Different letters denote significant differences according to one-way ANOVA (*p* < 0.05).

To explore the diurnal expression rhythms of *LcCOL* genes under natural photoperiod conditions, leaf samples were collected every 4 h over a 24 h cycle during the floral induction stage after autumn shoots maturation in litchi (Figure 5). Distinct diurnal expression patterns were observed among individual *LcCOL* family members. *LcCOL1* maintains high transcript levels from late night to throughout the daytime. *LcCOL2* showed dual expression peaks at dusk and dawn, with the dawn peak being significantly higher than the dusk one (*p* < 0.05). *LcCOL3* showed a single expression peak exclusively at dusk. The expression of *LcCOL4* peaked at approximately 4 h after nightfall and remained low near dawn and during the early morning (4 h after sunrise). *LcCOL5* shared a dual-peak expression pattern similar to *LcCOL2*, but displayed a markedly higher expression level at dusk than at dawn (*p* < 0.05). *LcCOL6* displayed elevated expression specifically at dawn and maintained low transcript abundance at all other time points. *LcCOL7* peaked at 8 h after nightfall and retained a relatively high level at dawn. Although *LcCOL8* shared the same peak time as *LcCOL7*, its expression declined sharply to an extremely low level by dawn. Collectively, these results reveal substantial divergence in the diurnal rhythmic expression of *LcCOL* genes, suggesting that these family members are subjected to distinct circadian clock-mediated regulatory mechanisms.

### 2.6. Overexpression of LcCOL7 and LcCOL8 Inhibits Flowering in Arabidopsis

**Figure 6:**
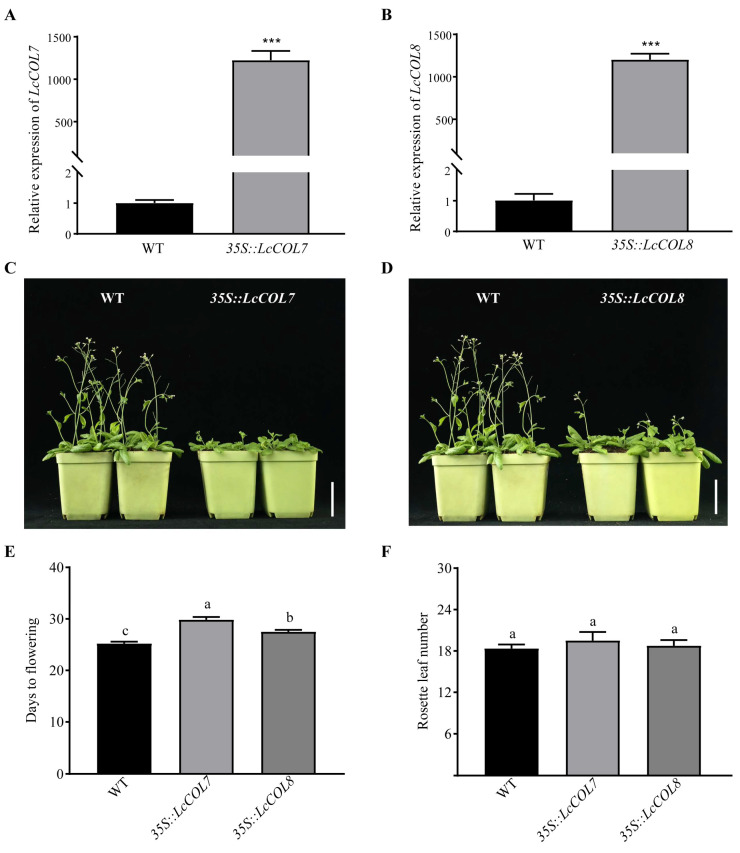
Overexpression of *LcCOL7* and *LcCOL8* in *Arabidopsis* inhibits flowering under long-day (LD) conditions. (**A**,**B**) Transcript levels of *LcCOL7* and *LcCOL8* in wild-type (WT) and transgenic lines. Error bars represent the standard error of the mean (*n* = 3). Asterisks (***) indicate significant differences (*p* < 0.001, Student’s *t*-test). (**C**,**D**) Representative flowering phenotypes of WT and *LcCOL7*/*LcCOL8* transgenic plants grown under LD conditions. Scale bar = 5 cm. (**E**,**F**) Statistical analysis of flowering time (days to flowering) and rosette leaf number at bolting of WT and transgenic plants. Error bars represent standard error of the mean (*n* = 20). Different letters above the columns denote significant differences (*p* < 0.05, one-way ANOVA).

Among the eight *LcCOL* family members, *LcCOL7* and *LcCOL8* exhibit the closest phylogenetic relationship with *AtCO.* To further characterize their biological functions, we heterologously overexpressed *LcCOL7* and *LcCOL8* in *Arabidopsis* driven by the constitutive 35S promoter. Stable transgenic *35S::LcCOL7* and *35S::LcCOL8* lines were successfully generated via hygromycin resistance screening. Transcript levels of *LcCOL7* and *LcCOL8* were subsequently quantified in wild-type (WT) plants and the corresponding transgenic lines to verify line validity (Figure 6A,B). Quantitative analysis confirmed that the expression levels of *LcCOL7* and *LcCOL8* were significantly elevated in their respective transgenic lines relative to WT plants (*p* < 0.001), indicating that the obtained overexpression materials were reliable and suitable for subsequent phenotypic analysis.

**Figure 7:**
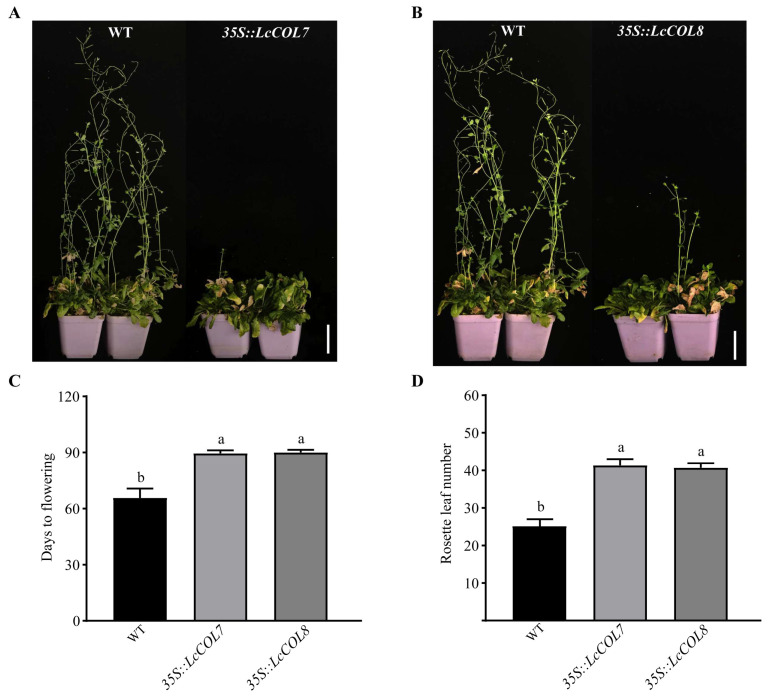
Over-expression of *LcCOL7* and *LcCOL8* delays flowering in *Arabidopsis* under short-day (SD) conditions. (**A**,**B**) Representative flowering phenotypes of WT and *LcCOL7*/*LcCOL8* transgenic plants grown under SD conditions. Scale bar = 5 cm. (**C**,**D**) Statistical quantification of flowering time (days to flowering) and rosette leaf number at bolting of WT and transgenic plants. Error bars represent standard error of the mean (*n* = 20). Different letters above the columns indicate significant differences (*p* < 0.05, one-way ANOVA).

To clarify the roles of *LcCOL7* and *LcCOL8* in flowering regulation, we evaluated the flowering phenotypes of *35S::LcCOL7* and *35S::LcCOL8* transgenic *Arabidopsis* plants. Bolting time and the number of rosette leaves at bolting were adopted as key flowering indices. Under LD conditions (Figure 6C–F, Supplementary Table S6), both transgenic lines exhibited a significantly delayed bolting day compared with WT plants (*p* < 0.05), while no obvious difference was observed in the number of rosette leaves at bolting. Under SD conditions (Figure 7, data in Supplementary Table S7), the flowering of the *35S::LcCOL7* and *35S::LcCOL8* lines was also markedly delayed, with an average flowering delay of more than 20 days. Additionally, transgenic plants produced significantly more rosette leaves at bolting than the WT control (*p* < 0.05). Collectively, these results indicate that both *LcCOL7* and *LcCOL8* function as flowering repressors in *Arabidopsis* by inhibiting floral transition, and this inhibitory effect is considerably stronger under SD conditions.

## 3. Discussion

In the present study, we identified eight *COL* homologs in litchi. Phylogenetic clustering divided these *LcCOL* genes into three distinct subgroups: Group I contains *LcCOL1*, *LcCOL2*, *LcCOL7*, and *LcCOL8*; Group II only harbors *LcCOL5*; and Group III includes *LcCOL3*, *LcCOL4*, and *LcCOL6*. Subsequent characterization of conserved motifs and protein domains further validated the accuracy of this phylogenetic grouping. There are 17 *COL* gene family members in *Arabidopsis*, including the key flowering regulator *CO*. By comparison, 16, 9, 33, 9, 15 and 10 *COL* genes have been reported in rice, barley, rapeseed, strawberry, pear and longan, respectively [4,5,6,7,8]. Such variation in gene copy number suggests that the *COL* family is ubiquitous across the plant kingdom and subject to evolutionary conservation. At the functional level, the Arabidopsis AtCO protein promotes floral transition specifically under LD conditions [9,14,15,16]. By contrast, AtCOL3 and AtCOL4, which also fall into Group I, serve as flowering repressors regardless of photoperiod under both SD and LD regimes [33,34]. In the LD woodland strawberry (*Fragaria vesca*), overexpression of *FvCO* accelerates flowering under SD conditions, while *FvCO* silencing delays flowering under LD conditions, confirming that FvCO acts as a flowering activator [6]. In rice, Hd1 upregulates *Hd3a* expression under SD conditions but suppresses it under LD conditions [22,25]. Both RcCO and RcCOL4 promote flowering in *Rosa chinensis*; however, their divergent expression patterns (*RcCO* is preferentially expressed under LD and *RcCOL4* is preferentially expressed under SD conditions) guarantee floral initiation under favorable growth conditions, enabling the day-neutral flowering habit of rose [10]. Taken together, these published findings reveal that COL proteins are deeply conserved central modulators of photoperiodic flowering pathway, yet their biological functions have undergone evolutionary diversification across plant lineages. Consistent with a previous report showing that overexpression of longan *DlCOL4* delays flowering time in transgenic *Arabidopsis* under LD conditions [8], our results demonstrated that heterologous overexpression of litchi *LcCOL7* and *LcCOL8* significantly delayed flowering time under both LD and SD conditions (*p* < 0.05). Furthermore, it is noteworthy that the flowering time of *LcCOL7*-overexpressing lines was significantly later than that of *LcCOL8*-overexpressing lines under LD conditions (*p* < 0.05), while no obvious difference in flowering time was observed between the two transgenic lines under SD conditions. This discrepancy implies that LcCOL7 and LcCOL8 may rely on distinct regulatory interactors or interfere with different endogenous AtCOL proteins to modulate flowering in *Arabidopsis* in response to different photoperiods. As mentioned in previous studies, numerous *COL* genes execute their biological functions in a photoperiod-specific manner [22,25,33]. Consequently, future research into how *COL* genes regulate floral transition in litchi will provide valuable insights for molecular genetic research and targeted molecular breeding in this economically important fruit crop.

We systematically analyzed *cis*-acting elements within the promoter sequences of *LcCOL* genes. The results revealed that the *LcCOL* promoters harbor numerous light-responsive and hormone-responsive elements. Notably, all eight *LcCOL* genes contain at least one copy of the G-box motif, a canonical light-responsive element. Previous work has demonstrated that G-box acts as a crucial binding site for core circadian clock PRR proteins; this motif is also detected in the promoters of *PRR9* and *CIRCADIAN CLOCK ASSOCIATED 1* (*CCA1*) [35]. PRR proteins bind to G-box via their CCT domains to precisely fine-tune the transcription of target genes at dawn [35]. The prevalence of light-responsive *cis*-elements in *LcCOL* promoters may therefore underpin their divergent diurnal expression rhythms. Furthermore, hormone-responsive elements are widely distributed across *LcCOL* promoters, including ABA-responsive elements (ABREs), SA-responsive elements (TCA-element), methyl jasmonate-responsive elements (TGACG-motif and CGTCA-motif), GA-responsive elements (P-box, GARE-motif, and TATC-box), and auxin-responsive elements (TGA-element and AuxRR-core). Existing evidence shows that ABA signaling components known as ABRE-Binding Factors (ABFs) directly interact with ABRE sequences and upregulate *CO* expression to accelerate flowering under LD conditions with drought stress [16,36]. Moderate drought treatment during floral induction is recognized as a critical environmental cue promoting flowering in litchi, although drought alone cannot fully substitute for the low temperature requirements for floral initiation [37]. The abundant ABRE regulatory elements in the promoters of functional genes *LcCOL7* and *LcCOL8* suggests a potential link between these elements and drought-induced floral transition in litchi. Consistent with our findings, multiple light-responsive elements (including G-box), ABRE sites and methyl jasmonate-responsive TGACG/CGTCA-motifs, were also detected in the promoters of rapeseed *BnCOL* family genes [5]. This indicates that the crosstalk between COL proteins and environmental or hormonal responses is evolutionarily conserved across plant species. Future research will aim to dissect the interplay between phytohormonal signaling and *LcCOL* transcription, and further characterize the biological roles of *LcCOL* genes in litchi adaptation to variable environmental conditions.

Our tissue expression profiling revealed that most *LcCOL* genes displayed higher expression levels in leaf buds and leaves compared to other tissues, while their expression remained generally low in floral tissues. Interestingly, the expression levels of *LcCOL7* and *LcCOL8* were significantly higher in female flowers compared to male flowers (*p* < 0.05). Litchi, a monoecious plant with unisexual flowers, exhibits asynchronous opening of male and female flowers. In this study, the flowering sequence of ‘Feizixiao’ litchi demonstrated a pattern where male flowers bloomed prior to female flowers, aligning with the role of LcCOL7 and LcCOL8 as flowering repressors. These observations suggest that the differential expression of *LcCOL7* and *LcCOL8* between male and female flowers may play a regulatory role in the staggered blooming of male and female flowers in litchi. In addition, the transcript abundance of *LcCOL7* and *LcCOL8* was significantly higher in young leaves than in mature leaves (*p* < 0.05). Previous studies have demonstrated that mature leaves serve as the primary tissue sensing low-temperature floral-inductive signals in litchi [38,39]. We therefore hypothesize that the elevated expression of *LcCOL7* and *LcCOL8* in young leaves may inhibit litchi floral transition. Given that our functional characterization of *LcCOL7* and *LcCOL8* was only performed in *Arabidopsis*, further in planta functional assays are required to validate their biological roles within litchi itself. Diurnal expression analysis under the natural photoperiod revealed that *LcCOL4*, *LcCOL7*, and *LcCOL8* share expression patterns similar to *AtCO*, with expression peaking at night under SD conditions [40]. Specifically, the transcript abundance of *LcCOL7* was significantly higher at dawn than at 4 h after sunrise (*p* < 0.05), whereas no such difference was observed for *LcCOL8*. This implies that these two functionally homologous genes may be modulated by distinct sets of circadian clock regulators. Litchi is regarded as a DN plant, a classification largely derived from the study showing that the ‘Kwai Mi’ cultivar is capable of flowering under both LD and SD conditions [30]. Even so, genotypic variation in photoperiod responsiveness across different litchi cultivars cannot be excluded. Unlike its Sapindaceae relative longan, it is difficult to induce off-season flowering and fruiting in litchi. In the Northern Hemisphere, floral induction in litchi occurs strictly during autumn and winter, followed by floral bud differentiation and fruit development in spring, with fruit ripening concentrated in late spring and summer. Such a constrained seasonal window for floral induction strongly suggests that this developmental program is governed by circadian rhythmic pathways. Future investigations evaluating photoperiodic responses across multiple litchi cultivars will help unravel the contribution of the photoperiod pathway to litchi flowering with more conclusive evidence.

## 4. Materials and Methods

### 4.1. Identification of COL Gene Family Members in Litchi

To identify CONSTANS-LIKE (COL) family members in litchi (*Litchi chinensis* Sonn.), we first retrieved genome and proteome sequences of litchi from the Sapindanceae Genome Database, 2025 updated version (https://www.sapindaceae.com, accessed on 4 March 2025). COL protein sequences of *Arabidopsis thaliana* were obtained from the TAIR database, TAIR10 (https://www.arabidopsis.org/, accessed on 4 March 2025). The AtCO and 16 AtCOL protein sequences were used as queries to identify all putative COL homologs in the litchi genome using BLASTp with an e-value of ≤1 × 10^−5^. Subsequently, the IntoPro database Version 101.0 (https://www.ebi.ac.uk/interpro/, accessed on 4 March 2025) was used to verify these *LcCOL* protein candidates containing B-box-type zinc finger and CCT domains. The LcCOL proteins’ fundamental physicochemical properties, including number of amino acids, molecular weight, isoelectric point, instability index, aliphatic index and grand average of hydropathicity were analyzed using the ExPASy online tool (https://www.expasy.org/, accessed on 25 March 2026).

### 4.2. Phylogenetic Analysis of COL Family Genes and Chromosomal Distribution of LcCOL Genes

All AtCOL and LcCOL protein sequences were employed to construct a maximum likelihood (ML) phylogenetic tree with 1000 bootstrap replicates using TBtools v2.476 [41]. The generated phylogenetic tree was visualized utilizing the online tool iTOL v7.6 (https://itol.embl.de/, accessed on 10 March 2026). To determine the chromosomal distribution of *LcCOL* genes, genome annotation files were obtained from https://data.mendeley.com/datasets/kggzfwpdr9/1 (accessed on 27 March 2026). Subsequently, the positions of the *LcCOL* genes were mapped utilizing TBtools.

### 4.3. Analysis of the Conserved Motifs, Conserved Domains and Gene Structures of LcCOLs

The conserved motifs within the protein sequences were identified utilizing the MEME Suite v5.5.9 (http://meme-suite.org/, accessed on 31 March 2026) with a set parameter of a maximum of 10 motifs. The NCBI Conserved Domain Database was used to examine the conserved domain architecture of LcCOLs. Gene structure information for *LcCOLs* was extracted from the litchi genome annotation file. Finally, TBtools [41] was used to visualize the conserved motifs, protein domain structures, and gene structures.

### 4.4. Analysis of cis-Acting Regulatory Elements in the Promoters of LcCOLs

To determine the type and quantity of cis-regulatory elements within the *LcCOL* genes, the promoter region, specifically the 2000 base pairs upstream of these genes, were initially extracted from the genome. These promoter sequences were subsequently submitted to the Plant Cis-Acting Regulatory Element (PlantCARE) database (accessed on 1 April 2026) to identify potential *cis*-elements. TBtools software was employed to visualize the distribution of these *cis*-elements in the promoters and to generate a heatmap depicting the number of *cis*-elements present in the *LcCOL* promoters.

### 4.5. Sampling Procedures for Litchi Tissues

For the analysis of tissue expression patterns, the ‘Feizixiao’ litchi cultivar was used as the study material. Various litchi tissues were collected at different developmental stages. During the shoot growth phase, samples of young leaves, mature leaves, leaf buds, and stem segments were collected. In the flowering phase, both flower buds and fully opened male and female flowers were harvested. During the fruit development phase, small fruits were collected at one week after anthesis. All samples were promptly flash-frozen in liquid nitrogen and stored at −80 °C for subsequent analysis. Experiments were conducted in three independent biological replicates.

### 4.6. Expression Patterns of LcCOL Genes Under Natural Photoperiod Condition

During the flowering induction stage, sampling was conducted in Chengmai County, Hainan Province (19.72° N, 110.19° E) on sunny days, specifically 5 and 6 December. These days featured a natural photoperiod consisting of approximately 11 h of light and 13 h of darkness, with temperatures ranging from 18 °C to 29 °C and relative humidity ranging from 60% to 90%. The sampling process commenced at dawn, around 7:00 a.m. Four mature leaves were collected from each tree every four hours, culminating in a total of six sampling events. All samples were promptly flash-frozen in liquid nitrogen and stored at −80 °C for subsequent analysis. Experiments were conducted using three independent biological replicates.

### 4.7. RNA Extraction and Transcriptional Level Analysis

The collected samples were finely ground in liquid nitrogen, and total RNA was subsequently extracted utilizing the Plant Total RNA Extraction Kit (DP441, Tiangen, Beijing, China). The extracted RNA was reverse-transcribed into complementary DNA (cDNA) using the Reverse Transcription Kit (G592, ABM, Vancouver, BC, Canada) to facilitate further transcriptional analysis. Relative quantitative analysis was conducted employing the SYBR Green method (11184ES, Yeasen Biotechnology, Shanghai, China) and a LightCycler^®^ 480 Real-Time PCR System (Roche, Basel, Switzerland). The *LcACTIN*/*LITCHI007623* gene served as the internal reference gene, with all quantitative PCR primers detailed in Supplementary Table S1. A two-step amplification protocol was implemented: initial denaturation at 95 °C for 2 min, followed by 40 cycles of denaturation at 95 °C for 10 s and annealing/extension at 60 °C for 30 s. Relative expression levels were determined using the 2^−△△Ct^ method with triplicate technical repeats.

### 4.8. Generation of Constructs and Transgenic Plants

To generate *LcCOL* overexpression constructs, the protein-coding sequences of *LcCOL7* and *LcCOL8* were amplified via PCR and subsequently cloned into the pCAMBIA1302 vector under the control of the Cauliflower mosaic virus (CaMV) 35S promoter. The resulting recombinant plasmid was introduced into Agrobacterium tumefaciens strain GV3101, and Arabidopsis plants were transformed using the floral dip method mediated by Agrobacterium. Transgenic plants were selected using 20 mg/L hygromycin B (No. A600230, Sangon Biotech, Shanghai, China) and successful Arabidopsis transformants were identified through quantitative real-time polymerase chain reaction (qRT-PCR) analysis. Homozygous T3 generation Arabidopsis plants were utilized for the observation of flowering phenotypes.

### 4.9. Flowering Phenotype Analyses

Sterilized homozygous T3 generation *Arabidopsis* seeds were sown on Murashige and Skoog (MS) medium and subjected to vernalization in darkness at 4 °C for 2 days. Subsequently, the seeds were cultivated at 22 °C under a photoperiod of 12 h of light and 12 h of darkness (12 h light/12 h darkness). After 7 days, the seedlings were transplanted into soil and grown under either long-day (LD, 16 h light/8 h darkness) or short-day (SD, 8 h light/16 h darkness) conditions. The number of days required for the primary inflorescence stem to reach 1 cm in height was recorded to determine the bolting or flowering time. Additionally, the number of rosette leaves present at the time of bolting was counted. Each experimental group consisted of twenty individual plants, and all experiments were conducted using three independent biological replicates.

### 4.10. Statistical Analyses

Statistical analyses were performed using IBM Statistics SPSS v 26.0. Differences in expression levels or phenotypes between samples were assessed using either Student’s *t*-test or one-way ANOVA followed by Dunnett’s multiple comparison test. Graphical representations were created using GraphPad Prism version 8.3.0 and Adobe Illustrator 26.0.

## 5. Conclusions

In this study, eight members of the *LcCOL* gene family were identified in litchi. Promoter sequence analysis revealed that *LcCOL* promoters contain abundant *cis*-elements related to light, hormone and stress responses, with G-box, ABRE and ARE motifs being the most highly enriched. Transcript profiling indicated that most *LcCOL* genes are predominantly expressed in leaf buds, leaves and young fruits. Under natural photoperiod conditions, *LcCOL1* exhibited sustained high expression from late night to through the daytime, while the remaining *LcCOL* members displayed nighttime expression peaks. Heterologous overexpression of *LcCOL7* and *LcCOL8* significantly delayed flowering in transgenic *Arabidopsis thaliana*, suggesting that these two genes function as flowering repressors.

## Figures and Tables

**Table 1:** Physicochemical properties of LcCOL proteins.

Protein Name	Gene ID	Number of Amino Acid	Molecular Weight/Da	Isoelectric Point	Instability Index	Aliphatic Index	Grand Average of Hydropathicity
LcCOL1	*LITCHI016333*	374	40,554.44	5.94	42.82	65.05	−0.307
LcCOL2	*LITCHI016841*	347	37,567.67	5.88	42.33	63.86	−0.475
LcCOL3	*LITCHI026370*	370	41,967.47	5.64	54.79	63.54	−0.772
LcCOL4	*LITCHI021516*	422	45,524.6	5.11	53.55	56.02	−0.479
LcCOL5	*LITCHI005542*	458	50,839.34	5.42	50.15	56.75	−0.838
LcCOL6	*LITCHI005661*	499	56,234.71	5.44	52.06	65.65	−0.638
LcCOL7	*LITCHI019305*	352	39,127.43	5.56	42.58	64.66	−0.603
LcCOL8	*LITCHI019307*	352	39,189.71	5.8	34.98	65.99	−0.574

## Data Availability

The primers used in this study are provided in the Supplementary Materials (Table S1). The statistics on the types and quantities of *cis*-elements associated with light-responsive, hormone-responsive, stress-responsive and developmental processes in LcCOL promoters are provided in the Supplementary Materials (Tables S2–S5, respectively). Statistical data on the flowering phenotypes of the *LcCOL7* and *LcCOL8* transgenic plants under LD conditions are provided in the Supplementary Materials (Table S6), and the statistical data on the flowering phenotypes under SD conditions are provided in the Supplementary Materials (Table S7).

## Supplementary Materials

The following supporting information can be downloaded at: https://www.mdpi.com/article/10.3390/plants15142139/s1, Table S1: Primers used in this study. Table S2: Statistics on the types and quantities of *cis*-elements associated with light response in *LcCOLs* promoters. Table S3: Statistics on the types and quantities of *cis*-elements associated with hormone response in *LcCOLs* promoters. Table S4: Statistics on the types and quantities of *cis*-elements associated with stress response in *LcCOLs* promoters. Table S5: Statistics on the types and quantities of *cis*-elements associated with plant development in *LcCOLs* promoters. Table S6: Statistical data of flowering phenotypes of *LcCOL7* and *LcCOL8* transgenic plants under LD conditions. Table S7: Statistical data of flowering phenotypes of *LcCOL7* and *LcCOL8* transgenic plants under SD conditions.

## Abbreviations

The following abbreviations are used in this manuscript:

ABA	Abscisic acid
ABRE	Abscisic acid-responsive element
AI	Aliphatic index
ANOVA	Analysis of variance
ARE	Anaerobic responsive element
CaMV	Cauliflower mosaic virus
CCA1	CIRCADIAN CLOCK ASSOCIATED 1
CDF	CYCLING DOF FACTOR
CO	CONSTANS
COL	CONSTANS-LIKE
COP1	CONSTITUTIVE PHOTOMORPHOGENIC 1
DN	Day-neutral
FKF1	FLAVIN-BINDING KELCH REPEAT F-BOX 1
FT	FLOWERING LOCUS T
GA	Gibberellin
GARE	Gibberellin-responsive element
GI	GIGANTEA
GRAVY	Grand average of hydropathicity
Hd1	Heading date 1
Hd3a	Heading date 3a
II	Instability index
JA	Jasmonic acid
LD	Long-day
ML	Maximum likelihood
NF-YB/NF-YC	NUCLEAR FACTOR Y B SUBUNIT/NUCLEAR FACTOR Y C SUBUNIT
PHYB	PHYTOCHROME B
pI	Isoelectric point
PRR9	PSEUDORESPONSE REGULATOR 9
qRT-PCR	Quantitative real-time polymerase chain reaction
RFT1	RICE FLOWERING LOCUS T 1
SA	Salicylic acid
SD	Short-day
SPA1	SUPPRESSOR OF PHYTOCHROME A 1
WT	Wild-type
