# Supplementary material for: LcCOL7 and LcCOL8 Negatively Regulate Plant Flowering Independent of Day Length
Source: Plants (Basel). 2026 Jul 10;15(14):2139. doi: 10.3390/plants15142139 (PMC13415198; doi:10.3390/plants15142139)
Supplement: Supplementary file 1 [file plants-15-02139-s001.zip › plants-4377344-supplementary.pdf]

**Supplementary Materials:**

**Table S1. Primers used in this study**

| Primers name | Sequence(5'-3') |
|---|---|
| LcCOL1-PCR-F | ATGGGAATCGACTTGGAAACCG |
| LcCOL1-PCR-R | CTAAAAGGATGGAACGACCCCG |
| LcCOL2-PCR-F | ATGGCTTCCAAGTTATGTGACTCC |
| LcCOL2-PCR-R | TTAAAAGGAGGGAACGACGCC |
| LcCOL3-PCR-F | ATGAACGAGGAAACGTTGACG |
| LcCOL3-PCR-R | TTAATGTTCAATCTTTGCAAAACGC |
| LcCOL4-PCR-F | ATGGGTTACATATGTGATTTTTGTGG |
| LcCOL4-PCR-R | TCAGTAGCTTCTGGTTTGGC |
| LcCOL5-PCR-F | ATGATTTCGAACAAAAATGTGGCC |
| LcCOL5-PCR-R | TCATTTGCCAATTAACAAGTTAGGA |
| LcCOL6-PCR-F | ATGGAGAAAATTTGTGAGTTCTGC |
| LcCOL6-PCR-R | TCACGTGATATCTTTAGTGTCTGA |
| LcCOL7-PCR-F | CCGTTAGTACAGCTCCCTCCC |
| LcCOL8-PCR-F | ACTACACCTTCCTCCCTTGCC |
| LcCOL7/8-PCR-R | TTAGAATGAAGGAACAATGCCATATCC |
| 1302-LcCOL7-NcoI-F | acgggggactcttgaccatggATGATGAAAGAAGAAAGCAGCG |
| 1302-LcCOL8-NcoI-F | acgggggactcttgaccatggATGATGAAAGAAGAGAGCAGCG |
| 1302-LcCOL7/8-SPeI-R | aagttcttctcctttactagtGAATGAAGGAACAATGCCATATCCG |
| LcCOL1-RT-F | AAAGTGTTTCATCGTCATCGCTTGA |
| LcCOL1-RT-R | CTGGGTCGCTTGGTTTGCAGTAG |
| LcCOL2-RT-F | GGGAACAGAACAGTTCAGGCACG |
| LcCOL2-RT-R | TGGCACCACTCCTACATCCAACG |
| LcCOL3-RT-F | TTGCCCATCTGTGAGCGAACTGT |
| LcCOL3-RT-R | CGAGAACCCATCAGCATCACCAT |
| LcCOL4-RT-F | GAATTGACAGCTTATTTGGGACA |
| LcCOL4-RT-R | CTTTGCGTTGAAACATAGGATTG |
| LcCOL5-RT-F | CACTCCCTCTTGTGCCAGAACTT |
| LcCOL5-RT-R | CTCAACGCCACTTTGGATTCACT |
| LcCOL6-RT-F | TCCAGCAAAGTGACAATCCAGTG |
| LcCOL6-RT-R | CACCTCCAAATAGTTCTTCAAAGTTCC |
| LcCOL7-RT-F | CGAGCGTGTCTTCGTTTGTG |
| LcCOL7-RT-R | GTATGGGAACTCGGTGGTGG |
| LcCOL8-RT-F | CAGTGACAGCATTGTTCCTGT |
| LcCOL8-RT-R | CCGTTGTAACTGTAAGCAGCTTTT |
| LcACT-RT-F | GTGGTTCTACTATGTTCCCTG |
| LcACT-RT-R | CTCGTCGTACTCATCCTTTG |
| AtACTIN2-RT-F | TGTGCCAATCTACGAGGGTTT |
| AtACTIN2-RT-R | TTTCCCGCTCTGCTGTTGT |

**Table S2. Statistics on the types and quantities of *cis*-elements associated with light response in LcCOLs promoters**

| Element type | LcCOL 1 | LcCO L2 | LcCOL 3 | LcCOL 4 | LcCOL 5 | LcCOL 6 | LcCOL 7 | LcCOL 8 | Total |
|---|---|---|---|---|---|---|---|---|---|
| G-box | 4 | 6 | 2 | 4 | 1 | 5 | 2 | 1 | 25 |
| TCT-motif | 0 | 2 | 1 | 0 | 2 | 3 | 4 | 2 | 14 |
| Box 4 | 0 | 0 | 4 | 3 | 2 | 1 | 1 | 1 | 12 |
| GT1-motif | 0 | 0 | 1 | 1 | 2 | 2 | 2 | 0 | 8 |
| I-box | 0 | 0 | 2 | 0 | 1 | 0 | 0 | 2 | 5 |
| AE-box | 0 | 1 | 1 | 0 | 0 | 0 | 0 | 3 | 5 |
| MRE | 1 | 0 | 0 | 1 | 1 | 1 | 0 | 0 | 4 |
| TCCC-motif | 0 | 0 | 1 | 1 | 0 | 0 | 1 | 1 | 4 |
| GATA-motif | 0 | 0 | 1 | 0 | 0 | 0 | 0 | 3 | 4 |
| AT1-motif | 0 | 0 | 0 | 0 | 2 | 1 | 0 | 0 | 3 |
| ATCT-motif | 0 | 1 | 0 | 1 | 0 | 0 | 1 | 0 | 3 |
| chs-CMA1a | 1 | 0 | 0 | 0 | 1 | 0 | 0 | 0 | 2 |
| ATC-motif | 2 | 0 | 0 | 0 | 0 | 0 | 0 | 0 | 2 |
| LAMP-element | 1 | 0 | 1 | 0 | 0 | 0 | 0 | 0 | 2 |
| ACE | 0 | 0 | 1 | 0 | 0 | 0 | 1 | 0 | 2 |
| chs-CMA2a | 0 | 0 | 0 | 0 | 0 | 0 | 0 | 1 | 1 |
| Pc-CMA2a | 0 | 0 | 0 | 0 | 1 | 0 | 0 | 0 | 1 |
| Sp1 | 1 | 0 | 0 | 0 | 0 | 0 | 0 | 0 | 1 |
| GA-motif | 1 | 0 | 0 | 0 | 0 | 0 | 0 | 0 | 1 |
| AAAC-motif | 1 | 0 | 0 | 0 | 0 | 0 | 0 | 0 | 1 |
| 3-AF1 binding site | 0 | 0 | 0 | 0 | 0 | 0 | 1 | 0 | 1 |
| GAP-box | 0 | 0 | 1 | 0 | 0 | 0 | 0 | 0 | 1 |

**Table S3. Statistics on the types and quantities of *cis*-elements associated with hormone response in LcCOLs promoters**

| Element type | LcCO L1 | LcCOL 2 | LcCOL 3 | LcCOL 4 | LcCOL 5 | LcCOL 6 | LcCOL 7 | LcCOL 8 | Total |
|---|---|---|---|---|---|---|---|---|---|
| ABRE | 2 | 2 | 1 | 2 | 0 | 3 | 3 | 3 | 16 |
| TGA-element | 0 | 1 | 1 | 1 | 0 | 0 | 0 | 0 | 3 |
| AuxRR-core | 0 | 1 | 0 | 0 | 0 | 0 | 0 | 0 | 1 |
| GARE-motif | 0 | 0 | 1 | 0 | 1 | 0 | 0 | 0 | 2 |
| P-box | 1 | 0 | 0 | 0 | 0 | 0 | 1 | 0 | 2 |
| TATC-box | 0 | 1 | 0 | 1 | 0 | 0 | 0 | 0 | 2 |
| as-1 | 0 | 0 | 0 | 0 | 1 | 0 | 0 | 0 | 1 |
| CGTCA-motif | 0 | 0 | 0 | 0 | 1 | 1 | 0 | 0 | 2 |
| TGACG-motif | 1 | 0 | 0 | 4 | 0 | 0 | 0 | 0 | 5 |
| TCA-element | 1 | 0 | 3 | 3 | 0 | 0 | 4 | 2 | 13 |

**Table S4. Statistics on the types and quantities of *cis*-elements associated with stress response in LcCOLs promoters**

| Element type | LcCOL 1 | LcCOL 2 | LcCOL 3 | LcCOL 4 | LcCOL 5 | LcCOL 6 | LcCOL 7 | LcCOL 8 | Total |
|---|---|---|---|---|---|---|---|---|---|
| O2-site | 1 | 3 | 0 | 0 | 0 | 0 | 1 | 1 | 6 |
| AT-rich sequence | 0 | 1 | 0 | 1 | 0 | 0 | 0 | 0 | 2 |
| AT-rich element | 0 | 1 | 0 | 0 | 0 | 0 | 0 | 0 | 1 |
| CAT-box | 0 | 1 | 0 | 0 | 0 | 0 | 0 | 1 | 2 |
| MBSI | 0 | 0 | 0 | 1 | 0 | 0 | 0 | 0 | 1 |
| AACA_motif | 0 | 0 | 0 | 0 | 1 | 0 | 0 | 0 | 1 |

**Table S5. Statistics on the types and quantities of *cis*-elements associated with plant development in LcCOLs promoters**

| Element type | LcCO L1 | LcCOL 2 | LcCOL 3 | LcCOL 4 | LcCOL 5 | LcCOL 6 | LcCOL 7 | LcCOL 8 | Total |
|---|---|---|---|---|---|---|---|---|---|
| ARE | 3 | 2 | 2 | 5 | 0 | 4 | 2 | 5 | 23 |
| MBS | 0 | 0 | 2 | 1 | 1 | 1 | 0 | 0 | 5 |
| TC-rich repeats | 0 | 0 | 1 | 1 | 1 | 0 | 0 | 1 | 4 |

**Table S6. Statistical data of flowering phenotypes of LcCOL7 and LcCOL8 transgenic plants under LD conditions**

| Genotype | Days to Flowering | Rosette Leaf Number |
|---|---|---|
| WT | 25.20±0.38c | 18.35±0.59a |
| *35S::LcCOL7* | 29.85±0.54a | 19.50±1.27a |
| *35S::LcCOL8* | 27.50±0.39b | 18.75±0.84a |

**Table S7. Statistical data of flowering phenotypes of LcCOL7 and LcCOL8 transgenic plants under SD conditions**

| Genotype | Days to Flowering | Rosette Leaf Number |
|---|---|---|
| WT | 65.72±5.01b | 26.56±2.38b |
| *35S::LcCOL7* | 89.50±1.61a | 41.38±1.62a |
| *35S::LcCOL8* | 90.00±1.41a | 40.70±1.72a |
